# Extracellular BAG3 is elevated in early diffuse systemic sclerosis

**DOI:** 10.1186/s40779-025-00628-w

**Published:** 2025-07-23

**Authors:** Paul Freedman, Margot De Marco, Alessandra Rosati, Liberato Marzullo, Nicoletta Del Papa, Maria Caterina Turco, Steven O’Reilly

**Affiliations:** 1 Biosciences Department, Durham University, Durham, DH1 3LE UK; 2 Department of Medicine, Surgery and Dentistry, University of Salerno, 84081 Baronissi, Italy; 3 Scleroderma Clinic, UOC Clinica Reumatologica, ASST Pini-CTO, Università Degli Studi Di Milano, Milan, Italy

**Keywords:** Systemic sclerosis, Bcl-2 associated athanogene-3 (BAG3), Cytokine, Lung disease, Stimulator of interferon genes (STING)

Dear Editor,

Systemic sclerosis (SSc) is an autoimmune connective tissue disease in which there are vascular abnormalities, inflammation, and fibrosis [1]. These 3 characteristics primarily affect the skin and lungs. Of all the autoimmune rheumatic diseases, SSc has the highest all-cause mortality rate, and the underlying pathogenic processes that mediate disease are still obscure, with wide differences in presentation and progression [2, 3]. It is believed that fibroblasts are involved in SSc pathogenesis by differentiating to myofibroblasts, which secrete copious amounts of extracellular matrix. Bcl-2 associated athanogene-3 (BAG3) is a multifunctional protein whose expression is extremely restricted but is highly expressed in cardiac and skeletal muscle tissue as well as multiple cancers. BAG3 belongs to a family of co-chaperones, and as such, is associated with a cellular stress response being positively regulated by heat shock factor 1. Given its pronounced anti-apoptotic function and its association with human cancers, including leukaemia and pancreatic cancer [4], it exerts a pro-survival effect via its anti-apoptotic role, which is associated with myofibroblasts. Most recently, it was demonstrated that extracellularly released BAG3 could alter fibroblasts’ motility and expression of α-smooth muscle actin, a key myofibroblasts marker, in the tumour microenvironment of fibrotic tumours. Furthermore, BAG3 was detected in the serum and skin of SSc patients [5]. Thus, we aimed to further examine the expression and role of BAG3 in early SSc.

Twenty patients with early diffuse SSc (defined at < 2 years from the first non-Raynaud’s symptom) were recruited, and serum was taken as a disease control, primary Sjögren’s syndrome (pSS) patients. All patients fulfilled the criteria for diffuse SSc, and all were treatment naïve. More details are presented in Additional file 1: Methods. The mean age of the SSc patients was 50 (range 35–66) years. The patient demographic data are shown in Additional file 1: Table S1. We used an established in-house enzyme-linked immunosorbent assay (ELISA) to quantify the level of BAG3 in these patients compared with 20 healthy controls (HCs) (age-matched). Serum BAG3 was significantly increased in SSc patients compared with HCs (Fig. 1a) [(33.80 ± 4.70) vs. (247.81 ± 30.02) pg/ml, *P* ≤ 0.0001; no difference between HCs and pSS; SSc vs. pSS was *P* ≤ 0.0001]. Comparing Scl-70-positive SSc patients with RNAPoll-II-positive (*n* = 2), the results showed that there was no statistically significant difference between Scl-70-positive patients [(253 ± 33.12) pg/ml] and RNAPoll-II-positive patients [(200.4 ± 30.40) pg/ml] (*P* = 0.6). However, Mann-Whitney *U* test with a small sample size, a definitive conclusion is hard to draw. No correlation between serum BAG3 and modified Rodnan Skin Score was found. Separating the SSc patients into those with or without interstitial lung disease (ILD), we observed that those with ILD had significantly elevated levels of BAG3 compared with those without ILD in Fig. 1b (*P* = 0.0002). Of those with ILD, there is no correlation between BAG3 concentration and diffusion capacity of the lungs for carbon dioxide (DLCO)% (*r* = − 0.5, 95% CI − 0.959 to 0.683, *P* = 0.39). However, this was a small subgroup with only 5 patients with ILD available a correlation may be present in a larger cohort.

**Fig. 1:**
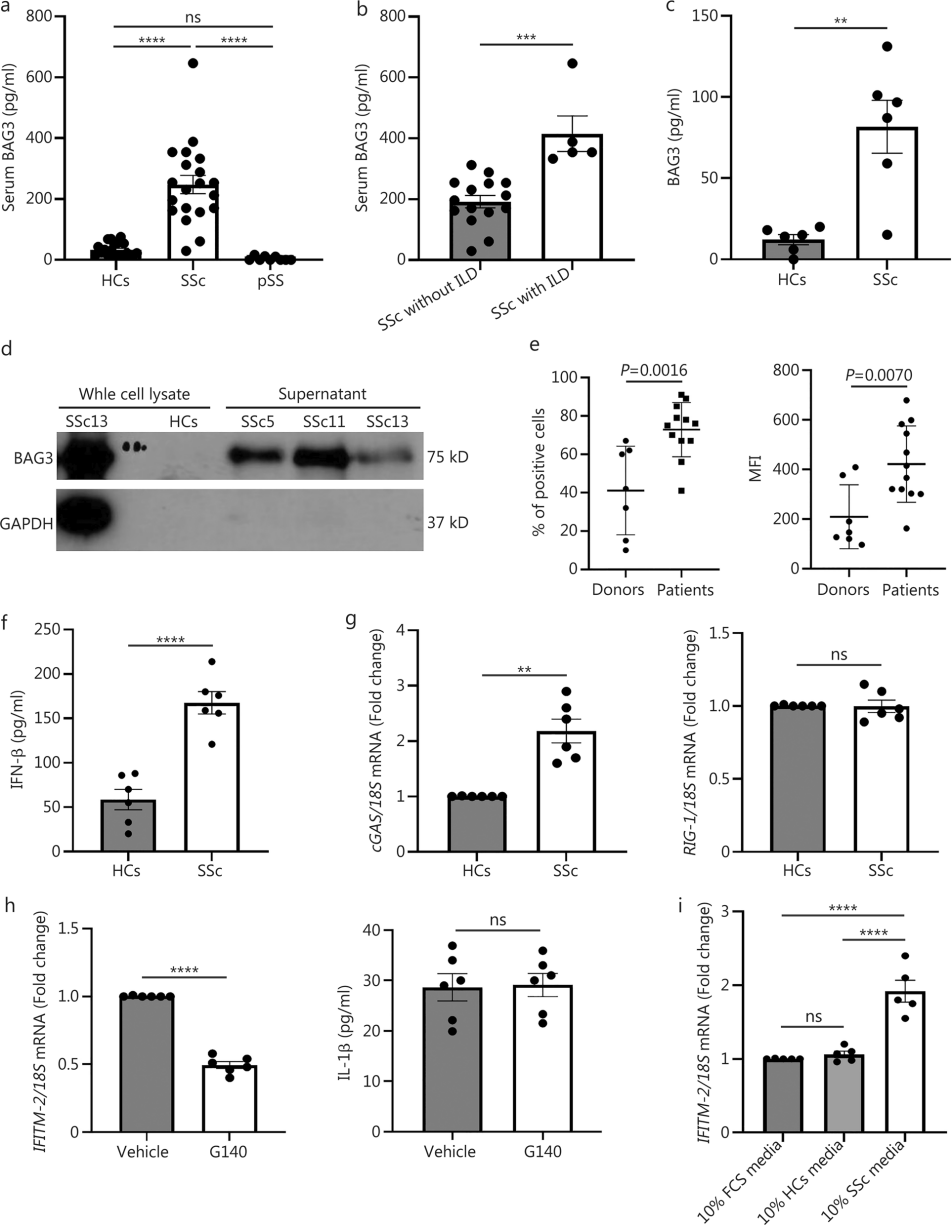
Elevated Bcl-2 associated athanogene-3 (BAG3) in diffuse systemic sclerosis (SSc) but not pSS. **a** Elevated serum BAG3 in SSc. Healthy controls (HCs) or SSc patient sera were quantified by ELISA for BAG3. ANOVA with Tukey’s multiple comparisons was used. Data are presented as the mean ± standard error of the mean (SEM), ^****^P ≤ 0.0001, ns non-significant; *n* = 20 for SSc patients and HCs, *n* = 9 for pSS patients. **b** Elevated serum BAG3 in SSc patients subdivided into those without and with interstitial lung disease (ILD). Students’ *t*-test was used. Data are presented as the mean ± SEM, ^***^P = 0.0002; *n* = 15 for SSc without ILD, *n* = 5 for SSc with ILD. **c** BAG3 was quantified by specific ELISA in cultured HC and SSc fibroblasts. Students’ *t*-test was used. Data are presented as the mean ± SEM, ^**^P = 0.0019; *n* = 6. **d** Western blotting of HCs and SSc patients of the whole cell lysate or supernatant. GAPDH is the cell loading control. **e** Interferon-induced transmembrane-2 (IFITM-2) was quantified by flow cytometry with a specific antibody. The left-hand panel is % of positive cells, and the right-hand panel is the mean fluorescence intensity (MFI). Data are presented as the mean ± SEM; *n* = 7 for HC and *n* = 12 for SSc. **f** Interferon-β (IFN-β) was quantified by ELISA in HCs or SSc fibroblasts. Students’ *t*-test was used. Data are presented as the mean ± SEM, ^****^P ≤ 0.0001; *n* = 6. **g** cGAS and RIG-I were quantified by quantitative RT-PCR. Data was normalised to 18S as the endogenous housekeeping gene, and data is shown as fold change compared to HCs fibroblasts. The Mann–Whitney U test was used. Data are presented as is the mean ± SEM, ^**^P = 0.0022, ns non-significant; *n* = 6. **h** SSc fibroblasts were treated with G140 (10 μmol/L) or DMSO for 24 h, after which IFITM-2 and IL-1β were quantified by quantitative RT-PCR. Data were normalised to 18S as the endogenous housekeeping gene, and were shown as fold change compared to vehicle-treated fibroblasts. Students’ *t*-test was used. Data are presented as the mean ± SEM, ^****^P ≤ 0.0001, ns non-significant; *n* = 6.** i** Normal HC fibroblasts were incubated with standard 10% FCS-containing media, HCs 10% serum-containing media or SSc 10% containing media for 24 h after which IFITM-2 was quantified by quantitative RT-PCR. Data was normalised to 18S as the endogenous housekeeping gene, and data is shown as fold change compared to normal media 10% FCS. ANOVA was used. Data are presented as the mean ± SEM, ^****^P ≤ 0.0001, ns non-significant; *n* = 5. ELISA enzyme-linked immunosorbent assay, RT-PCR reverse transcription-polymerase chain reaction, FCS fetal calf serum

Six patients’ skin biopsies were taken from the lesional skin, and fibroblasts were explanted and cultured. We tested the extracellular levels of BAG3 in these dermal fibroblasts, and found significantly elevated levels of BAG3 (Fig. 1c; *P* = 0.0019). This was further validated by Western blotting using a specific antibody to BAG3 in SSc dermal fibroblasts. Figure 1d revealed that BAG3 is highly elevated in the media from SSc skin fibroblasts. This is highly novel and could elicit a response both in fibroblasts to evoke myofibroblasts and in fibrocytes. Fibrocytes are known to be elevated in SSc [6].

Recently, the putative receptor for BAG3 was identified as interferon-induced transmembrane-2 (IFITM-2). After binding to IFITM-2, BAG3 can activate downstream signalling pathways, including the mediators p38 and PI3K [7]. We therefore quantified the levels of IFITM-2 on the surface of the fibroblasts from HCs or SSc patients. The findings demonstrated that SSc patients’ fibroblasts have significantly elevated levels of the BAG-3 receptor IFITM-2 (Fig. 1e). This suggests that SSc fibroblasts have intrinsically elevated IFITM-2 expression, possibly induced by type I interferon (IFN). To that end, we quantified the expression of IFN-β in the SSc fibroblasts. There was a significantly elevated endogenous release of IFN-β from the SSc fibroblasts compared to HCs fibroblasts (Fig. 1f; *P* ≤ 0.0001). Because type I IFN is mediated via activation of the cyclic GMP-AMP synthase (cGAS)-stimulator of interferon genes (STING) pathway after DNA activation, we examined the expression of cGAS. Figure 1g showed the elevation of cGAS in SSc fibroblasts compared to HCs fibroblasts (twofold increase, *P* = 0.0022). However, the RNA intracellular sensor retinoic acid inducible gene-I (RIG-I), which can also trigger type I IFN release, was not elevated in SSc fibroblasts (Fig. 1g). Because of the endogenous constitutive release of IFN, we used a specific cGAS inhibitor, which will block the IFN release and determined the expression of IFITM-2. Figure 1h revealed that inhibiting cGAS with G140 reduced the IFITM-2 expression compared to vehicle control-treated cells (*P* ≤ 0.0001). This was concurrent with significant IFN-β reduction (vehicle-treated vs. G140, 170.70 vs. 68.83 pg/ml, *P* ≤ 0.0001). However, an irrelevant cytokine independent of cGAS-STING, interleukin (IL)-1β, was unaffected (Fig. 1h; *P* = 0.37). This implicates cGAS-STING-IFN signalling in IFITM-2 constitutive expression. Finally, we collected the serum from 5 patients with SSc ILD, added this serum to the culture media, and then placed this media on HCs fibroblasts alongside HCs serum diluted or standard culture media with 10% fetal calf serum (FCS) and quantified IFITM-2 levels. Figure 1i indicated that SSc serum led to a significant induction of IFITM-2 compared to HCs serum and standard culture media (*P* ≤ 0.0001). It has been demonstrated in previous studies that SSc patients have a prominent IFN signature [8, 9] and correlate with disease features, and we suggest that IFN in the serum induces IFITM-2 upregulation. Furthermore, we took SSc patients’ serum and pre-incubated the fibroblasts with anifrolumab, to block IFN signalling or an isotype control or the IL-6 inhibitor tocilizumab. It is shown that anifrolumab attenuated the induction of IFITM-2, but the isotype control or IL-6 blockade did not (Additional file 1: Fig. S1). This strongly suggests that IFN is driving the induction of IFITM-2 in fibroblasts.

In conclusion, we demonstrate elevated BAG3 in early diffuse SSc sera and release by SSc skin fibroblasts. Additionally, the receptor for BAG3 IFITM-2 is significantly elevated on SSc fibroblasts and is regulated by IFN via the cGAS-STING pathway, suggesting that SSc patients’ sera can induce IFITM-2 likely through type I IFN. This is consistent with the study in which IFN is upregulated, and recently, perturbation of cGAS has been described in fibroblasts from ILD-SSc patients [10]. Altogether, our data suggest BAG3 as a potential therapeutic target of SSc therapy. Whether serum BAG3 could be used as a prognostic indicator of disease progression warrants further investigation, especially those SSc patients with ILD.

## Supplementary Information

**Additional file 1.** Methods. **Table 1** Patient demographics. **Fig. S1** IFN drives upregulation of IFITM-2 in fibroblasts.

## Data Availability

Not applicable.

## Abbreviations

BAG3: Bcl-2 associated athanogene-3
cGAS: Cyclic GMP-AMP synthase
HC: Healthy controls
IFITM-2: Interferon-induced transmembrane-2
IFN-β: Interferon-β
IL: Interleukin
ILD: Interstitial lung disease
pSS: Primary Sjögren’s syndrome
STING: Stimulator of interferon genes
SSc: Systemic sclerosis
