# Supplementary material for: Extracellular BAG3 is elevated in early diffuse systemic sclerosis
Source: Mil Med Res. 2025 Jul 23;12:37. doi: 10.1186/s40779-025-00628-w (PMC12285001; doi:10.1186/s40779-025-00628-w)
Supplement: Supplementary file 1 — Additional file 1. Methods. Table 1 Patient demographics. Fig. S1 IFN drives upregulation of IFITM-2 in fibroblasts. [file 40779_2025_628_MOESM1_ESM.pdf]

Methods

Twenty patients with early diffuse systemic sclerosis (SSc) were involved in the study, recruited in the rheumatology department at Newcastle University. Patients were defined as early diffuse SSc, defined as < 2 years since the first non-Raynaud's symptom. All patients fulfilled the American College of Rheumatology (ACR) criteria for a diagnosis of diffuse systemic sclerosis, and full informed consent was provided by the patients involved. Primary Sjögren's syndrome (pSS) patients met the 2002 American-European Consensus Group criteria and the ACR-EULAR criteria. Demographic data is in the **Additional file 1: Table S1**. Healthy controls (HCs) were free from disease and age, and gender matched.

The study has full ethical approval with the local research ethics committee (REC) with approval no REC/13/NE/0089 and followed the Declaration of Helsinki guidelines. HCs were age and gender-matched (n = 20). A total of 15 ml of blood was drawn from each donor's arm, and serum was isolated by centrifugation at 2000× g for 15 min. The serum was frozen immediately at -150 ℃ until thawed for downstream analysis.

Six early diffuse SSc patients' whole skin biopsies were taken in the affected skin on forearms by dermal punch biopsy (4 mm^3) from the affected area. These were then placed into tissue culture and minced with a scalpel, and fibroblasts were left to grow out. Cells were cultured in DMEM (Sigma, UK) supplemented with 10% fetal calf serum (FCS) and L-glutamine (2 mmol/L), penicillin (100 U/ml), and streptomycin (100 μg/ml) (all Sigma, UK) in an incubator at 5% CO_2 at 37 °C. Dermal fibroblasts from control donors were isolated identically and treated in the same way. All fibroblasts used for experiments were at passage 3 and tested for mycoplasma.

Cyclic GMP-AMP synthase (cGAS) was inhibited with the specific inhibitor G140 (10 μmol/L) (Invivogen, UK) and was incubated in SSc cells or vehicle control (0.01% DMSO) for 24 h, after which time the cells were lysed for RNA isolation. Supernatants were removed for interferon (IFN)-β and interleukin (IL)-1β expression.

Enzyme-linked immunosorbent assay (ELISA)

Serum was thawed and used in an ELISA for BAG3 which had been used previously. The supernatant was removed from diffuse SSc or HC fibroblasts, and BAG3 was measured using the above ELISA. IFN-β and IL-1β were quantified by specific ELISA according to the manufacturer's instructions, Quantikine ELISA R&D Systems United Kingdom (catalogue no: DIFNBO IFN and DLB50 IL-1β).

Quantitative reverse transcription-polymerase chain reaction (RT-PCR)

RNA was isolated from healthy and SSc skin fibroblasts and healthy controls after mechanical disruption using TRIzol and 1 μg RNA converted to cDNA using Nanoscript 2 reverse transcriptase (Primer Design Ltd, Southampton, UK) Q-RT-PCR was performed using specific primers and SYBR™ green (Sigma, UK) all data was normalized to the housekeeping gene 18S and relative differences computed using the delta Ct method. No template control was run as a control. Data is shown as fold change compared to HCs. Primers cGAS F: 5'-CTCCACGAAGCCAAGACCTC-3', Rev: 5'-GCGGCTGAGCTTCAACTTCT-3'. Retinoic acid-inducible gene (RIG)-I F: 5'-AGAGCACTTGTGGACGCTTT-3', Rev: 5'-TCAGCAACTGAGGTGGCAAT-3'. Interferon-induced transmembrane-2 (IFITM-2) F: 5'-CCTTGACCTGTATTCCACT-3', Rev: 5'-GCCATTGTAGAAAAGCGT-3'. 18S F: 5'-CGA ATG GCTCATTAAATCAGTTATGG-3', Rev: 5'-TATTAGCTCTAGAATTACCACAGTTATCC-3'. Data is normalized to 18S internal housekeeper.

Patient and control serum experiments

Five early diffuse SSc sera samples were taken and five HC sera and diluted to a final concentration of 10% (volume/volume) or normal 10% FCS in standard culture media and added to 6-well plates in duplicate with HC dermal fibroblasts and incubated for 24 h. After 24 h in the different media compositions, the cells were lysed for q-PCR to assess IFITM-2 expression.

In some experiments, before the addition of the SSc serum, the fibroblasts were incubated with anifrolumab, IgG1 antibody as an isotype control, and tocilizumab, all at 2 μg/ml for 1 h, after which

the SSc 10% containing sera was added. After 24 h the cells were lysed for qPCR for IFITM expression normalized to 18S ($n = 3$).

IFITM-2 flow cytometry

Fibroblasts from Ssc-patients and healthy donors were harvested with Non-Enzymatic Cell Dissociation Solution (ATCC 30-2103) and incubated with binding buffer (PBS 1×/10% decomplemented fetal bovine serum (FBS)/0.1% NaN_3) and FcR Blocking Reagent (Miltenyi Biotec, Germany; 130-059-901) following the manufacturer's instructions. Then, cells (1×10^6/ml) were resuspended in a binding buffer (15 min at 4 °C) and incubated with FITC-conjugated anti-IFITM2 5D11B9 murine mAb (30 min at 4 °C). An unrelated murine mAb was used as a negative control. Cells were then washed three times with washing buffer (PBS 1×/2% decomplemented FBS/0.1% NaN_3), centrifuged (10 min at 300× *g*), and resuspended in binding buffer for flow cytometry analysis by FACSVerse Flow Cytometer (BD Biosciences, UK). 7-AAD (#420404, BioLegend, UK) was used for the exclusion of non-viable cells in the flow cytometric assay. Data is shown as % IFITM-2 positive cells and mean fluorescent intensity.

Western blotting

Cells were cultured in DMEM w/o serum for 16 h. Supernatants were then collected and subjected to sequential centrifugations: 1) 10 min at 300× *g*; 2) 10 min at 2000× *g*; 3) 30 min at 10,000× *g*. After each centrifugation, pellets (cells, dead cells) were discarded, and the supernatant was transferred for the following step. Supernatants were mixed with 3V acetone and incubated overnight at -20 °C, and then centrifuged at 20,000× *g* for 15 min. Precipitated proteins were resuspended in 20 μl of 1× Laemmli buffer (containing SDS and beta-mercaptoethanol for reduction) and loaded on 10% SDS-PAGE at 120 V. Western blotting analysis was performed by using an HRP-conjugated rabbit polyclonal anti-BAG3 antibody (1:1000 dilution in milk 10%, overnight, at 4 °C). GAPDH was probed by a monoclonal antibody (sc-32233, Santa Cruz Biotechnology, USA) as an intracellular protein control.

Statistical analysis

Data was analyzed using either a One-way ANOVA or a Students' *t*-test, or a Mann-Whitney *U* test, with a significance set at $P \leq 0.05$, analyzed with Prism™ software. The data presented are the mean ± SEM. For correlation analysis, Pearson's two-tailed correlation was performed.

Table S1 Patient demographics

SSc patient number	Age (year)	Sex	Autoantibodies	CRP (mg/L)	mRSS	Treatment	ILD	DLCO%	Cardiac disease
Patient 1	42	F	Scl-70	-	23	None	N	85	None
Patient 2	54	F	Scl-70	-	16	None	N	82	None
Patient 3	51	F	RNA-polIII	-	26	None	N	89	None
Patient 4	37	F	Scl-70	-	11	None	N	73	None
Patient 5	52	F	Scl-70	-	12	None	N	79	None
Patient 6	41	M	Scl-70	-	11	None	N	89	None
Patient 7	42	F	Scl-70	-	18	None	N	91	None
Patient 8	57	M	Scl-70	-	22	None	N	75	None
Patient 9	35	F	Scl-70	-	7	None	N	82	None
Patient 10	49	F	Scl-70	-	20	None	N	76	None
Patient 11	63	F	Scl-70	-	16	None	N	74	None
Patient 12	37	F	Scl-70	-	15	None	N	78	None
Patient 13	66	F	Scl-70	-	17	None	Y	60	None
Patient 14	49	F	Scl-70	-	19	None	Y	57	None
Patient 15	55	F	RNA-polIII	-	15	None	Y	52	None
Patient 16	55	F	Scl-70	-	21	None	Y	50	None
Patient 17	48	F	Scl-70	-	11	None	Y	52	None
Patient 18	46	F	Scl-70	-	21	None	N	88	None
Patient 19	59	F	Scl-70	-	17	None	N	81	None
Patient 20	63	F	Scl-70	-	25	None	N	90	None
pSS 1	56	F	ANA	1.4	NA	HCQ	ND	ND	None
pSS 2	43	F	ANA	3.4	NA	None	ND	ND	None
pSS 3	35	F	SSA	0.7	NA	None	ND	ND	None
pSS 4	39	F	ANA	1.1	NA	None	ND	ND	None
pSS 5	46	F	ANA	2.5	NA	None	ND	ND	None
pSS 6	29	F	ANA	1.6	NA	None	ND	ND	None
pSS 7	47	F	ANA	0.8	NA	HCQ	ND	ND	None
pSS 8	44	F	ANA	1.2	NA	None	ND	ND	None
pSS 9	61	F	ANA	1.4	NA	None	ND	ND	None

NA not applicable, *ND* not done, *CRP* C-reactive protein, *mRSS* modified Rodnan skin score, *ILD* interstitial lung disease, *DLCO* diffusion capacity of the lung for carbon monoxide, *HCQ* hydroxychloroquine, *pSS* primary Sjögren's syndrome, *SSc* systemic sclerosis, *F* female, *M* male, *ANA* antinuclear antibodies

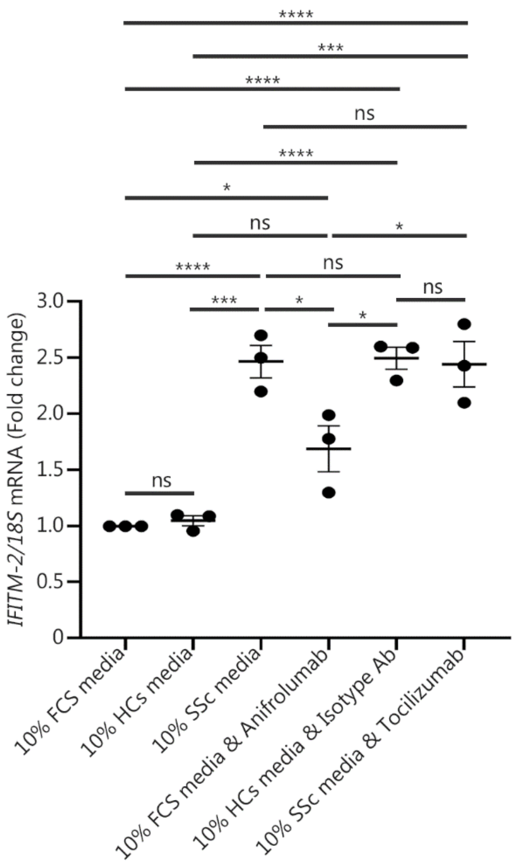

Fig. S1 IFN drives upregulation of IFITM-2 in fibroblasts. Normal HC fibroblasts were incubated with standard 10% FCS-containing media, HC 10% serum-containing media, or SSc 10% containing media, or pretreated with anifrolumab, isotype control, or tocilizumab for 1 h before SSc serum-containing media for 24 h after which IFITM-2 was quantified by quantitative RT-PCR. Data was normalised to 18S as the endogenous housekeeping gene, and data is shown as fold change compared to normal media 10% FCS. ANOVA with Tukey's corrections was used. Data are presented as the mean and standard error of the mean (SEM). $^{*}P \leq 0.05$, $^{***}P \leq 0.001$, $^{****}P \leq 0.0001$, ns non-significant. $n = 3$. IFN interferon, IFITM-2 interferon-induced transmembrane-2, BAG3 Bcl-2 associated athanogene-3, HC healthy controls, FCS fetal calf serum, SSc systemic sclerosis, RT-PCR reverse transcription-polymerase chain reaction
